# The lifecycle of the Ebola virus in host cells

**DOI:** 10.18632/oncotarget.18498

**Published:** 2017-06-15

**Authors:** Dong-Shan Yu, Tian-Hao Weng, Xiao-Xin Wu, Frederick X.C. Wang, Xiang-Yun Lu, Hai-Bo Wu, Nan-Ping Wu, Lan-Juan Li, Hang-Ping Yao

**Affiliations:** ^1^ State Key Laboratory for Diagnosis and Treatment of Infectious Diseases, The First Affiliated Hospital, College of Medicine, Zhejiang University, Hangzhou, China; ^2^ Collaborative Innovation Center for Diagnosis and Treatment of Infectious Diseases, Hangzhou, China; ^3^ Department of Bioengineering, Erik Jonsson School of Engineering and Computer Science, The University of Texas at Dallas, Dallas, TX, USA

**Keywords:** Ebola virus, EBOV proteins, EBOV lifecycle

## Abstract

Ebola haemorrhagic fever causes deadly disease in humans and non-human primates resulting from infection with the Ebola virus (EBOV) genus of the family *Filoviridae*. However, the mechanisms of EBOV lifecycle in host cells, including viral entry, membrane fusion, RNP formation, GP-tetherin interaction, and VP40-inner leaflet association remain poorly understood. This review describes the biological functions of EBOV proteins and their roles in the lifecycle, summarizes the factors related to EBOV proteins or RNA expression throughout the different phases, and reviews advances with regards to the molecular events and mechanisms of the EBOV lifecycle. Furthermore, the review outlines the aspects remain unclear that urgently need to be solved in future research.

## INTRODUCTION

*Filoviridae* are from the order *Mononegavirales*, include Ebola virus (EBOV), Marburg virus (MARV) and Cuevavirus [1, 2], which are single-stranded, negative-sense RNA viruses that exhibit unique heterogeneous filamentous structure. Filovirus was first reported and named Marburg virus in 1967 during an outbreak of viral haemorrhagic fever (HF) in Frankfurt (Germany) and Belgrade (Yugoslavia) [3]. In 1976, EBOV was determined to be the cause of outbreaks of viral HF in the Sudan and Congo [4]. Five different species of EBOV have since been established: 1) Zaire virus (Zaire EBOV); 2) Sudan virus (Sudan EBOV); 3) Bundibugyo virus (Bundibugyo EBOV); 4) Taï Forest virus (Taï Forest EBOV); and 5) Reston virus (Reston EBOV) [5]. Filovirus HF is transmitted directly via contact with bodily fluids from infected patients or other species (e.g., gorillas and chimpanzees) [6]. Infection is characterized by high levels of inflammatory cytokines, coagulation disorders, poor immune response and lymphopenia, which results in septic shock andmultiorgan failure finally [7–9].

**Figure 1:**
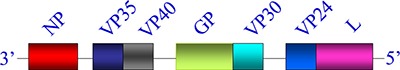
EBOV genome The genes are depicted as boxes: nucleoprotein (NP), viral protein (VP) 35, matrix protein VP40, glycoprotein (GP), VP30, VP24, and polymerase protein (L).

EBOV is composed of seven genes coding at least ten proteins from the 3′ leader to the 5′ trailer: 1) nucleoprotein (NP); 2) viral protein 35 (VP35); 3) VP40; 4) glycoprotein (GP); 5) soluble GP (sGP); 6) Δ-peptide; 7) ssGP; 8) VP30; 9) VP24; and 10) polymerase (L) [10, 11] (Figure 1). Until now, biological functions of these proteins and their roles in the EBOV lifecycle in host cells have been largely clear. NP forms a large complex with VP30 and VP35 that encapsulates the viral genome, represents the polymerase cofactor, and involve in synthesizing viral RNAs [12, 13]. VP35 is involved in the formation of the viral nucleocapsid and L cofactor, dissociates NP-RNA oligomers, and releases the genomic RNA from NP-RNA complexes for further replication [13, 14]. In addition, it is implicated in regulating the interferon response to EBOV and modulating other aspects of the host immune response [15, 16]. VP40 has an important role in the maintenance of viral integrity and aggregation at the cell membrane for virion budding and egress [17, 18]. GP1 and GP2 are two subunits of the glycoprotein (GP_1,2_) which produced by the cleavage of a precursor (GP0) obtained by the translation of an mRNA derived from an editing process of the primary transcript that codify the GP soluble form [19]. Moreover, there is an alternate transcription editing site in the GP gene, which leads to the expression of additional proteins, including the soluble GP (sGP), the Δ-peptide, and the small soluble GP (ssGP) [11, 20]. GP1 is responsible for interacting with one or more cellular receptors, GP2 contains a fusion loop that is critical for membrane fusion, while sGP is supposed leads to immune subversionan and acts as a decoy for antibodies directed against GP_1,2_ [21–23]. The Δ-peptide is suggested to regulates filovirus entry as its expression limits infection on filovirus-permissive cells [11, 24]. Yet, the function of ssGP in viral pathogenicity remains unclear. VP30 is an activation of transcription factor involved in -ssRNA packaging and nucleocapsid construction [25]. VP24 is suggested to block IFN-α/β/γ signalling, interact with the endosomal traffic protein, and is required for a fully functional nucleocapsid [26, 27]. Although extensive progress has been made in the knowledge of the mechanisms throughout the EBOV lifecycle in host cells, there are still several aspects remain poorly understood. In this review, we discuss the unravelled mechanisms and the outstanding questions regarding the EBOV lifecycle in host cells and the advanced strategies for further research.

### Viral entry

#### Attachment

The detailed mechanisms of EBOV attachment are currently partially explored. EBOV infects a wide variety of mammals, which complicates the identification of cellular proteins required for viral attachment. Previous studies have demonstrated that EBOV attachment on target cells is mediated by the binding of the transmembrane virus envelope GP1 to cell surface factor (s) [28]. GP1 has three distinct domains: 1) the receptor binding domain (RBD); 2) the glycan cap; and 3) the heavily O-linked glycosylated mucin-like domain (MLD) [29]. RBD is responsible for interacting with one or more cellular receptors. The glycan cap could protect the receptor binding sites from antibodies, and interacts with the internal fusion loop of GP2 that is critical for GP2-mediated membrane fusion for preventing pre-mature fusion events [30]. Glycosylation is extensive in GP1 MLD, it probably shield GP receptor binding sites from immune recognition and contributes to GP maturation and function, although not required for virus entry [31, 32]. X-ray crystallography structure showed that the glycosylated glycan cap and MLD are surround the RBD, coated a thick layer of oligosaccharides. This conformation probably benefit the need to truncate the transmembrane and certain glycosylation sites in order to achieve crystallization [33]. To date, there are several factors that have been reported as EBOV receptors or co-receptors. The C-type lectin family contains carbohydrate recognition domains (CRDs) that bind the glycan cap, since GP is highly glycosylated with several types of sugar side chains [34]. The family, including asialoglycoprotein receptor (ASGP-R), dendritic cell-specific ICAM-3-grabbing nonintegrin (DC-SIGN), human macrophage galactose and acetylgalactosamine-specific C-type lectin (hMGL), and lymph node sinusoidal endothelial cell C-type lectin (LSECtin/CLEC4G), have all been shown to interact with EBOV GP and facilitate viral attchment. For example, ASGP-R is specifically expressed in hepatocytes and is reported to bind and benefit endocytosis of GP containing a terminal galactose, DC-SIGN and its homolog, L-SIGN were found to recognize high-mannose carbohydrate moieties and mediate attachment to the cell surface [35, 36]. hMGL expression on immature DCs and macrophages was reported to recognize galactosyl residues and act as an attachment factor for EBOV and MARV [37]. LSECtin/CLEC4G was described to bind N-acetylglucosamine of GP and enhance Filovirus infection [38].

Then, the Tyro3 protein kinase (TAM) family, including Axl, Dtk, and Mer, which widely expressed in many cell types, span the plasma membrane and contain intracellular tyrosine kinase domains, are facilitate EBOV GP-dependent attachment [39, 40]. In contrast, Shimojima M *et al*. [41] reported Tyro3 family-independent entry of GP-pseudotyped murine leukemia virus (MLV) in Vero-E6 cells, which demonstrated the interaction of other unknown factors and the complexity of the filovirus entry mechanism. Meanwhile, T-cell immunoglobulin mucin domain (TIM), including TIM-1 and TIM-4 are demonstrated to bind the receptor binding domain of the EBOV GP [42, 43]. TAM, TIM-1 and TIM-4 target phosphatidyl-serine (PtdSer), which is exposed on the outer leaflet of the filovirus membrane, strengthening an interplay promoting efficient attachment [44, 45]. Later, β1 integrins, responsible for extracellular matrix attachment is thought to stimulate endosomal proteases required for EBOV transduction and increase EBOV GP-mediated pseudovirion entry [46, 47]. However, no direct interaction has been found between GP and the β1 integrin family.

In addition, folate receptor-α (FR-α) was reported to be a co-receptor in binding cells that expressed MARV or EBOV GP, mediated syncytia formation triggered by GP, and facilitated cellular entry of the virus [28]. However, other papers questioned the role of FR-α as an important factor for Ebola virus entry, and presumed it was an additional alternative factor, as FR-α highly expressed cells were not conferred susceptibility to EBOV GP compared with FR-α insufficient cells [48, 49].

#### Uptake

Filoviruses virions are uptaked into host cells involve different endocytic pathways. First, the internalization mechanisms were controversial over a period of time as clathrin-dependent and caveolin-dependent uptakes have been shown to occur [34, 43, 50]. However, later data supported that clathrin and caveolin-mediated endocytosis was not important for EBOV entry, but macropinocytosis and other factors on the host cell and virus particle size were critical factors [51–53]. Other studies using pseudotype viruses packaged with EBOV proteins asserted that the endocytic pathway of EBOV entry was dependent upon the endocytic enzymes cathepsin B/L and cholesterol, a major component of caveolae and lipid-rafts [43, 52]. Several GTPases, such as RhoB, Rac1, and CDC42, have been implicated in endocytosis and play an important role in EBOV GP-dependent transduction [54]. Low pH was shown helpful for GP-mediated membrane fusion. Acidic conditions have no direct effect but pH-dependent cathepsin activity to affect GP-mediated fusion [54, 55]. Once EBOV GP is cleaved by cathepsin, acidic conditions directly induce conformational changes in cleaved GP that lead to fusion. It's confirmed that cell-cell fusion exhibits a maximum at pH 5.7; the pH-dependence of fusion in later is eliminated when EBOV GP is cleaved, and the extent of fusion was independent of pH [56]. The studies indicated the pH-dependence of fusion is solely due to the ability of cathepsin to cleave EBOV. After the virus has been internalized into the endosome, fusion induced by cleaved GP is fundamentally independent of pH, which indicated an unidentified host cell factor critical for filovirus entry is sensitive to an acidic pH [56].

Taken together, the mechanisms of EBOV entry have been partially characterized. There is an ongoing debate as the appropriateness of the background used for pseudotype viruses and that EBOV probably infect different cell types via other mechanisms. The details and mutual connections of those identified factors are currently poorly understood as there is a missing link to the mechanisms that have been found to date. Therefore, several of these molecules lack effective integration and require further identification.

### Uncoating and fusion

Following endocytosis, the next steps consist of the uncoating and fusion of the viral membrane with the endosomal membranes. Precursor GP (GP0) is cleaved by the host enzyme, furin in the Golgi apparatus, resulting in GP1, GP2, and additional proteins, including sGP, Δ-peptide, and ssGP [11, 57]. GP2 is critical for membrane fusion, as it's composed of five domains: 1) a fusion loop; 2) an N-terminal heptad repeat region; 3) a C-terminal heptad repeat region; 4) a transmembrane region; and 5) a short cytoplasmic tail [58]. The glycan cap of GP1 can interact with the internal fusion loop of GP2 to restrict the availability of the fusion peptide and prevent premature fusion events [30]. The fusion loop, which contains a core hydrophobic sequence of 16 amino acids, is thought to insert into host endosomal membranes and initiate membrane fusion process [59, 60].

However, unknown enzymes trigger and accelerate this fusion process. it's considered that an endosomal/lysosomal factor (e.g., lysosomal thiol reductase) which inhibited by cysteine protease inhibitors and restricted by a low pH, triggers the fusion events [61]. What's more, 23 enzymes of the ARF family of GTPases involved in membrane traffic machinery, especially the small GTPase Rab7 related to the late endosomes was reported to accelerate virion fusion [62, 63]. Thus, further work is necessary to identify the factors required to trigger filovirus GP-mediated fusion. Following the insertion of the GP2 fusion loop into the host membrane, the GP2 trimeric heptad repeats (HR) recombine and form a transmembrane six-helix bundle containing three HR1 and HR2 domains. This bundle triggers an opening through the membrane, as described elsewhere [64]. Then the viral RNA and associated proteins can be released into the host cell cytoplasm for replication.

Niemann-Pick C1 (NPC1) is a ubiquitous protein with 12 transmembrane helices domain, termed as “sterol-sensing domain” (SSD), and two luminal domains, resides primarily in the late endosomes and lysosomes. The biological function of NPC1 is mainly as cholesterol transporter and re-distribution to cellular membranes, as the membranes is endosomal-receptor of EBOV and crucial for EBOV membrane fusion and entry [65–68]. The helical structure core of NPC1 contains two extended loops and are surrounded by severalβstrands. The crystal structure revealed that NPC1 domain C utilizes the two loops to engage a hydrophobic cavity at the head of the primed GP (GPcl). After conformational changes, the uplift of the short helix in the loop helps to release the N-terminal portion of the internal fusion loop, then triggering the membrane fusion [65, 68]. The SSD domain includes a two-way cavity open to both the endosomal lumen and the luminal leaflet of the lipid bilayer, and is large enough to accommodate one cholesterol molecule, which is important for NPC1 transports cholesterol across the lipid bilayer [68]. Meanwhile, the activity of the transport is regulated by the cholesterol concentration of the endosomal bilayer, cells disrupted by an NPC1 inhibitor or lacking NPC1 exhibited resistance to EBOV infection [66, 68]. So, NPC1 could be a critical hub between external cholesterol uptake and internal biosynthetic pathways. The details of how NPC1 influences EBOV invasion has yet to be further research.

### Transcription and replication

Similar to other −ssRNA viruses, the RNA genome of EBOV is encapsulated by NP and further form a ribonucleoprotein (RNP) complex together with RNA-dependent RNA polymerase (RdRp) [69]. After entry into the cytoplasm and membrane fusion, the RNP is released from the virion and serves as the template. Complementary positive stranded RNA (cRNA) is produced in the form of an RNP, and then generates viral genomic RNA to be packaged into the virions. It is reported that in the entire viral replication cycle of a -ssRNA virus, the genomic length viral RNA (cRNA or viral genomic RNA) is only present in the form of an RNP that either serves as a template for RNA synthesis or is packaged in the virions [70, 71]. So, the correct RNP formation and function is a key step for the transcription, replication, and assembly for −ssRNA viruses. However, the molecular mechanism of EBOV RNP formation is largely unclear.

NP is composed of a N-terminus, C-terminus, and NP core domain (NP core) in the centre, which possesses an N-lobe and C-lobe to clamp an RNA binding groove [70]. The N- and C-terminal extend in a tetrameric structure to reach the RNA-binding groove, contribute to NP oligomerization in RNP formation and binding with RNA [71]. However, the VP35 N-terminal peptide binds to a hydrophobic site on the NP C-terminal domain with high affinity and specificity, inhibits NP oligomerization and releases RNA from NP-RNA complexes *in vitro* [14]. The crystal structure of EBOV VP35 reveals that it contains a coiled-coil domain, forms a tetramer state in solution, contributes to the oligomeric states and variations in RNA binding preferences, and benefits it's connection with the blunt-ended RNA termini in a cooperative manner [72, 73]. Yet, the driving force that directs the VP35 peptide to release RNA from the RNP remains largely unknown. It is thought that NP oligomerization and simultaneous RNA binding at the RNP complex might provide the necessary force to displace the VP35 peptide [74]. Moreover, it's demonstrated that the VP35 N-terminal peptide is responsible for preventing premature NP oligomerization and RNA binding by maintaining the protein in an NP-VP35 complex, and reversing the oligomerization of RNA-free NP oligomers, but has no effect on RNA-bound NP oligomers, which providing insight into NP's role as a part of the viral RNA synthesis machinery [75, 76]. However, further details and mechanisms have yet to be firmly established.

### Assembly and budding

Assembly of viral particles begins with the formation of nucleocapsids which accumulate in the perinuclear region and are transported to the budding sites at the plasma membrane. Throughout this processes, GP, VP24, NP, and VP40 proteins play different roles.

GP protein is synthesized in the endoplasmic reticulum (ER) as a precursor and transported along the classical secretory pathway from the ER via the Golgi apparatus to the plasma membrane. Precursor GP is processed by the acylation, oglycosylation, and maturation of N-glycans, and finally undergoes proteolytic cleavage by furin [36, 77, 78]. Acylation is another posttranslational modification of viral GP, involved in particle formation, including virus assembly and budding. After those processes, GP is partially recruited to the late endosome to meet with VP40 for assembly and budding [79].

VP24 protein has been proved a secondary matrix protein and minor component of virions and contributing to virion assembly [27, 80]. Silence of VP24 RNA resulted in a reduction in the number of released virions, but viral transcription and replication were not affected, implicating a role for VP24 in viral assembly and/or budding [81]. However, the detailed mechanisms surrounding the necessity of VP24 for assembly and budding are largely unknown.

The N terminus of NP protein shoulders NP-NP and NP-RNA interaction, whereas the C terminus changes with the NP-VP40 interaction [82, 83]. At the core of the nucleocapsid, NP helices are thought to physically interact with VP40 via the 50 C-terminal amino acids, and be incorporated into the VP40-induced VLPs [80]. It has been proved NP assembles into helical tubes, forms a nucleocapsid-like structure with VP35 and VP24, then migrate to the cell surface via microtubules mediated by VP40, and is finally incorporated into virions through an NP-VP40 interaction [84, 85]. This process is essential for nucleocapsid transport to the plasma membrane and incorporation into virions. In addition, the flexibility of the NP-NP interaction in oligomer formation allows RNP to be packaged into viral particles with higher structures and density [86, 87]. These results deepen our understanding of NP functionality in assembly and budding. More details regarding the self-assembly of helical tubes and the transmission process should be explored for the development of antiviral compounds.

VP40 is the most abundant viral protein located under the viral envelope, plays a vital role in maintaining structural integrity and maturation of the EBOV virion [88, 89]. VP40 contains two differently folded domains [i.e., N-terminal (NTD) and C- terminal (CTD)] [90]. In cytoplasm, the NTD hydrophobic interface of VP40 forms homodimers through contact with some cellular proteins including mammalian ubiquitin ligase (Nedd4/Rsp5), Tsg101, and Vps4, the protein-protein interaction causes translocation of the VP40 dimers to the plasma membrane [91, 92].

In budding, the interaction of VP40 and inner leaflet is one of the major processes. The N-terminal domain of VP40 constitutes oligomerization whereas the C-terminal domains are a flexible hydrophobic loop [93]. It's indicated that VP40 contains both electrostatic and hydrophobic components which are associated with plasma membrane phosphatidylserine (PS), VP40 binds PS-containing membranes with nanomolar affinity, while PS regulates VP40 localization and oligomerization on the inner leaflet of the plasma membrane [94, 95]. The rearrangement of the flexible hydrophobic loop induces the penetration and docking of the PS, and allows VP40 to lock into the membrane [96]. However, information is still lacking regarding how VP40 associates with the inner leaflet and further induces the orchestrated processes. The precise mechanisms are still unknown, although some peripheral proteins have been shown to be involved in the decrease of the desolvation penalty associated with hydrophobic membrane insertion [97, 98].

Meanwhile, the interaction of GP2 and tetherin is another major processes in budding. Tetherin is an IFN-a-induced, cell-surfaceprotein-based tether which can induces virion retention on the cell membrance [79, 99, 100]. GP2 contains a glycan cap and hydrophobic membrane spanning domain (MSD) that is suggested to play a considerable (but not the sole determinant) role in tetherin antagonism [101, 102]. However, how tetherin precisely induces virion retention, as well as the mechanism by which the GP glycan cap and MSD antagonize the antiviral activity of tetherin remain unknown.

As VP40 has many different locations within host cells, including the inclusions, late endosome, nucleocapsids, and MVBs. It is thought that VP40 may be transported to the site of budding either associated with nucleocapsid structures or with cellular membranes [103]. For example, VP40 is accumulated in the late endosome in high amounts for oligomerization and the formation of the regular arrays of VP40, myosin 10 could change the localization of intra-filopodia motility and release VP40-induced VLPs [104, 105]. However, there was no direct evidence of an interaction between myosin 10 and VP40. Therefore, VP40 has many complex roles in the processes of assembly and budding, although more detail is required with further study.

Filoviral budding, has not only been detected on the plasma membrane but on intracellular membranes of MVBs and late endosomes as well [106]. Intracellular viral particles might serve as a source of infectious units that can be delivered by exosomes combined with another signal-dependent process upon cell-to-cell contact. The cell-to-cell contact is supposed to promote EBOV GP-mediated infection, and increase the local concentration of retroviral pseudovirions and EBOV VLPs [107]. This would result in relatively high MOIs, thus enhancing infection and spread. As reports of exosomes in the EBOV lifecycle are limited, further investigation is required.

**Figure 2:**
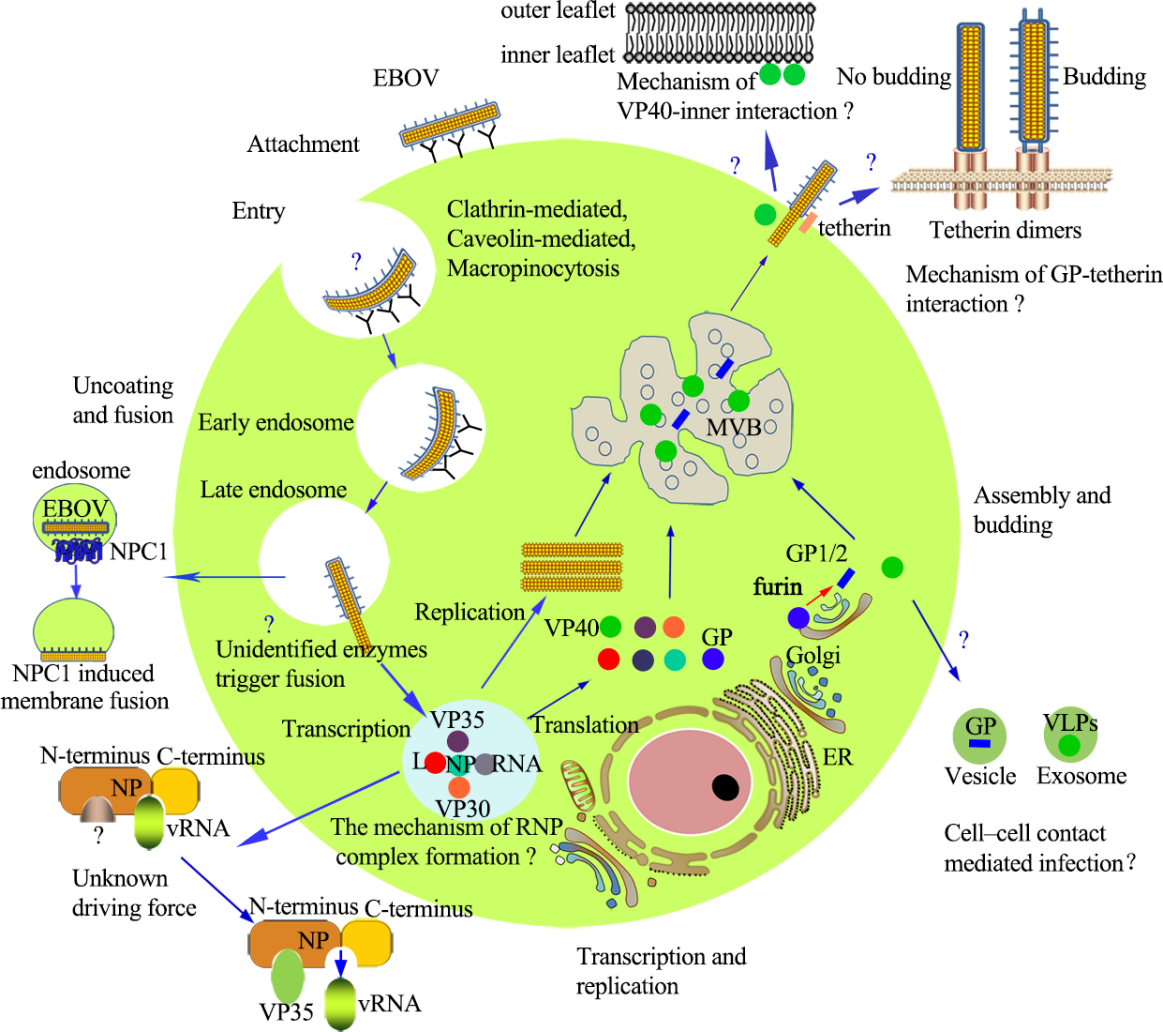
EBOV lifecycle Entry: EBOV enters cells via binding to receptors or co-receptors, in the macropinocytosis pathway, although debate still exists. Uncoating and fusion: In the endosome, proteolytic cleavage, and other unknown factors trigger uncoating of nucleocapsids; NPC1 induces the fusion of EBOV and cellular membranes. Transcription and replication: Viral mRNA is transcribed via the viral polymerase, and the viral proteins are subsequently translated. Replication of the viral genome is present in the form of RNP. The molecular mechanism of RNP and the mechanism by which VP35 releases RNA from RNP remain unknown. Assembly and budding: Assembly is initiated by the nucleocapsids which accumulate in the perinuclear region, and are then transported to the budding sites at the plasma membrane. Budding: Occurs at the plasma membrane, intracellular membranes of the MVBs and late endosomes. VP40 and GP play critical roles in the budding process. Abbreviations: ER: endoplasmic reticulum; MVB: multivesicular body; NPC1: Niemann-Pick C1.

In brief, glorious progress has been made in the mechanisms throughout the EBOV lifecycle in host cells. However, there are still several aspects remain poorly understood. The EOBV lifecycle is presented vividly in Figure 2.

## CONCLUSIONS

In summary, although considerable amounts of researches have uncovered the mechanisms of the EBOV lifecycle, there are still several aspects remain for further investigation (Table 1). EBOV entry into the cells is initiated by the interaction of the viral GP with receptors on the surface of host cells, and then internalized via macropinocytosis pathway. In uncoating and fusion, GP1 binds the endosome via RBD, and GP2 guides fusion via the fusion loop. Several host enzymes which remain to be fully characterized are regarded to catalyse the reaction. This is a challenge for researchers as there are abundant enzymes involved within host cells. Regarding replication, the key step is when VP35 releases RNA from the NP-RNA complexes by inhibiting NP oligomerization. However, the structural details and molecular mechanisms of EBOV RNP, and the dynamic of VP35 releasing RNA from RNP have not been completely defined. During assembly and budding, GP2 antagonizes the anchoring of tetherin via unarticulated mechanisms; VP40 regulates viral budding by associating with the inner leaflet of the plasma membrane with unknown detailed mechanisms. Therefore, the aspects that remain unclear in the EBOV lifecycle is waiting for profound research.

**Table 1:** The clarified, related, and unclear mechanisms of the EBOV lifecycle

Lifecycle	Clarified and Related Mechanisms	Unclear Mechanisms
Entry	GP-dependent, receptors or co-receptors (e.g., FR-α, integrin ß1, lectins, Tyro3, NCP1) induced endocytosis or micropinocytosis.	Unincorporated entry methods, unidentified proteins or receptors, un-illuminated pathways in different cell lines.
Uncoating and Fusion	GP-mediated, host enzyme cooperation	Unidentified enzymes trigger and accelerate the process.
Transcription and Replication	RNP (i.e., NP, VP30, VP35, L, RNA) complex-mediated.	The molecular mechanism of RNP is unclear. The driving force directing the VP35 peptide to release RNA from RNP is not clear.
Assembly and Budding	GP-tetherin interaction, VP24-induced correct assembly, NP-related nucleocapsid transport and the incorporation into virions, and VP40-inner leaflet association	How does GP antagonise tetherin? How does NP complete its function? How does VP24 contribute to assembly and budding? What is the mechanism used to control VP40 oligomerization? How does myosin-10 influence the localisation of intra-filopodia motility release? How does VP40 associate with the inner leaflet?
